# Effects of Flavin-Goethite Interaction on Goethite Reduction by *Shewanella decolorationis* S12

**DOI:** 10.3389/fmicb.2019.01623

**Published:** 2019-07-18

**Authors:** Gang Zhao, Enze Li, Jianjun Li, Fei Liu, Xunan Yang, Meiying Xu

**Affiliations:** ^1^ Guangdong Provincial Key Laboratory of Microbial Culture Collection and Application, Guangdong Institute of Microbiology, Guangdong Academy of Sciences, Guangzhou, China; ^2^ State Key Laboratory of Applied Microbiology Southern China, Guangzhou, China

**Keywords:** flavin, goethite, bioreduction, adsorption, *Shewanella decolorationis* S12

## Abstract

Flavin mononucleotide (FMN) and riboflavin are structurally similar flavins, except for the presence of a phosphate group on the FMN molecule. They are used by a variety of electroactive bacteria as extracellular electron shuttles in microbial Fe reduction and inevitably interact with Fe (hydr)oxides in the extracellular environment. It is currently unknown whether flavin/Fe (hydr)oxide interaction interferes with extracellular electron transfer (EET) to the mineral surface. In this study, we found that the goethite reduction rate was lower when mediated by FMN than by RF, suggesting that FMN was less effective in shuttling electrons between cells and minerals. Nevertheless, the phosphate group did not prevent the FMN molecule from accepting electrons from bacterial cells and transferring electrons to the mineral. Results of adsorption experiment, attenuated total reflectance (ATR) Fourier transform infrared (FTIR) spectroscopy, and bacterial attachment trend analyses showed that FMN exhibited strong adsorption on goethite surface by forming phosphate inner-sphere complex, which prevented bacterial cells from approaching goethite. Therefore, the interaction between FMN and goethite surface may increase the distance of electron transfer from bacterial cells to goethite and result in lower EET efficiency in comparison to those mediated by riboflavin. To our knowledge, these data reveal for the first time that the interaction between flavin and Fe (hydr)oxide affect flavin-mediated electron transfer to mineral surface and add a new dimension to our understanding of flavin-mediated microbial Fe reduction processes.

## Introduction

Microbial reduction of insoluble Fe(III) (hydr)oxides to soluble Fe(II) form is a ubiquitous redox process in natural environments (Weber et al., 2006; Byrne et al., 2015; Venkidusamy et al., 2018). It plays an important role in the geochemistry of soils and sediments, leading to migration of nutrients, dissolution of minerals and weathering, degradation of organic matter, and the mobilization or immobilization of heavy metal ions (Moore et al., 2002; Shi et al., 2016). Dissimilatory Fe-reducing bacteria, such as *Shewanella* and *Geobacter* species, are widely distributed in aquatic, terrestrial, and subsurface environments (Lovley, 1995; Dong et al., 2003; Yan et al., 2008; Byrne et al., 2015). They can transfer electrons to the mineral surface by direct contact or by using flavins [e.g., flavin mononucleotide (FMN) and riboflavin] as extracellular electron shuttles (von Canstein et al., 2008; Shi et al., 2016). The latter approach has been shown to be faster for reduction of Fe(III) (hydr)oxides (von Canstein et al., 2008; Ross et al., 2009; Huang et al., 2018). FMN and riboflavin as extracellular electron shuttles can be secreted by many bacteria (such as *Shewanella oneidensis* strain MR-1, *Shewanella decolorationis* strain S12, and *Geobacter sulfurreducens*) (von Canstein et al., 2008; Kotloski and Gralnick, 2013; Okamoto et al., 2014; Yang et al., 2017). In addition, several bacterial species have been found to be able to use FMN or riboflavin as an extracellular electron shuttle (Fuller et al., 2014; Light et al., 2018). Low concentrations of FMN and riboflavin have also been detected in pore water and water column profiles from a coastal marine basin, suggesting that FMN and riboflavin may be common components of extracellular Fe(III) reduction in natural environments (Monteverde et al., 2018).

To function as an extracellular electron shuttle, FMN or riboflavin first establishes contact with the surface of the bacterial outer membrane and insoluble Fe(III) (hydr)oxides. FMN and riboflavin molecules are known to interact with the hemeproteins such as MtrC and OmcA on the bacterial outer membrane to form flavin-hemeprotein complexes, resulting in a much higher microbial current generation than that of free flavin (Okamoto et al., 2013, 2014). Xu et al. (2016) reported that flavin-dependent extracellular electron transfer from *Shewanella* to electrodes was mediated mainly by outer-membrane hemeproteins-bound flavins. FMN have also been found to act as a cofactor for the hemeprotein to promote electron transfer from a porin-cytochrome complex embedded liposomes, a synthetic model of the *Shewanella* outer membrane, to iron oxides (Wang et al., 2015). The interactions between flavins and c-type cytochromes occur near the hemes, and riboflavin molecule may bind to heme 7 in OmcA (Paquete et al., 2014; Hong and Pachter, 2016). On the other hand, FMN and riboflavin inevitably interact with insoluble Fe (hydr)oxides once they are secreted into the extracellular environment. However, the interaction between flavins and Fe (hydr)oxides remains largely unmeasured. It is currently unknown whether the interaction between flavin and Fe (hydr)oxide affects extracellular electron transfer from bacterial cell to Fe (hydr)oxides.

Therefore, in this study, we investigated the effect of flavin-iron (hydr)oxide interaction on microbial Fe reduction. FMN and riboflavin, which differ in one phosphate group, were chosen as electron shuttles, while goethite, one of the most common Fe (hydr)oxides in nature (Cornell and Schwertmann, 2003), was used as the electron acceptor. *Shewanella decolorationis* strain S12, which can utilize FMN and riboflavin to mediate extracellular electron transfer, was selected to reduce goethite (Yang et al., 2017). Kinetics of goethite reduction by *S. decolorationis* S12/FMNH_2_/RFH_2_, and that of flavin reduction by *S. decolorationis* S12 were compared. The interfacial behavior of flavins and bacterial cells on the goethite surface were also evaluated.

## Materials and Methods

### Chemicals and Mineral

4-(2-hydroxyethyl) piperazine-1-ethanesulfonic acid (HEPES) and sodium formate were purchased from Sigma-Aldrich. FMN and riboflavin were purchased from Aladdin Chemistry Co. Ltd. Goethite was synthesized according to Parfitt and Atkinson (1976). The zeta potential and particle size of goethite before and after the adsorption of flavins were determined by using a Zetasizer (Nano ZS; Malvern Instruments Ltd.). The tested concentrations of goethite and flavin were ~1,125 and 100 μM, respectively. BET surface area of goethite was determined by N_2_ BET adsorption.

### Bacteria and Growth Conditions

*Shewanella decolorationis* strain S12^T^ (CCTCCM203093T = IAM 15094^T^) was available in the preserved form in our laboratory. All strains were grown aerobically to the log phase in standard Luria-Bertani (LB) medium. The cells were then harvested, washed three times with HEPES buffer (30 mM, pH 7.0), and suspended for subsequent use.

### Bioreduction of Flavin Mononucleotide and Riboflavin

FMN or riboflavin (10 μM, final concentration) was injected into a quartz cuvette sealed with a rubber stopper and deoxygenated by purging with N_2_ for 5 min. Deoxygenated cell suspension and sodium formate (electron donor) were injected into the quartz cuvettes to obtain a final concentration of ~10^7^ cells ml^−1^ and 10 mM, respectively. The total volume of the aqueous phase in quartz cuvettes was 4 ml. Fluorescence (excitation, 263 nm; emission, 522 nm) was monitored every 5 min using a PerkinElmer LS 45 fluorescence spectrometer.

### Reduction of Goethite by FMNH_2_ and RFH_2_

FMNH_2_ and RFH_2_ (the reduced forms of FMN and riboflavin) were prepared by incubating FMN or riboflavin with *S. decolorationis* S12 (~10^7^ cells ml^−1^) in HEPES containing sodium formate (10 mM). Compared with the chemical reduction (Shi et al., 2012, 2013), microbial reduction rates of FMN and riboflavin are slower. However, no endogenous flavins were detected in the treatment with only *S. decolorationis* S12 added during FMNH_2_ and RFH_2_ preparations (2 days) and the culture filtrate (0.2 μm) cannot reduce goethite. The culture containing FMNH_2_ or RFH_2_ was filtered inside an anaerobic glove box and injected into deoxygenated goethite suspension. The concentrations of reduced flavin and goethite were ~22 and ~1,125 μM, respectively. At desired time intervals, the Fe(II) (0.5 M HCl-extractable) was determined using Ferrozine analysis (Lovley and Phillips, 1987).

### Bioreduction of Goethite in the Presence of Flavin Mononucleotide and Riboflavin

Bioreduction experiments were conducted in 8 ml serum bottles crimp-sealed with Teflon-faced rubber stoppers. FMN or riboflavin (final concentrations 0, 1, 10, and 100 μM) solution was equilibrated with goethite suspension (final concentration ~1,125 μM) for 4 h. *S. decolorationis* S12 and sodium formate (final concentrations of ~10^7^ cells ml^−1^ and 10 mM) were then spiked into serum bottle to obtain a final volume of 5 ml. The solution was deoxygenated by bubbling with N_2_ for 20 min. The bottles were then placed in an incubator at 28°C and taken out at regular intervals to determine the total Fe(II) concentration in the samples. All the samplings and Fe(II) analyses were performed anaerobically.

### Batch Sorption, Attenuated Total Reflectance-Fourier Transform Infrared Experiments, and Two-Dimensional Correlation Spectroscopy (2D-COS) Analysis

Samples in triplicates were prepared in 8-ml serum bottles by mixing goethite (final concentration ~11.25 mM) and FMN or riboflavin (final concentrations ranging from 0 to 100 μM). A relatively high concentration of goethite was used to demonstrate the amount of flavin absorbed on goethite. Bottles were equilibrated at 28°C for 4 h and centrifuged at 20,000 × *g* for 10 min. The supernatant was filtered through a 0.45-μm polytetrafluoroethylene filter and analyzed by high-performance liquid chromatography using a fluorescence detector (excitation, 450 nm; emission, 520 nm). The concentration of FMN or riboflavin adsorbed onto goethite was calculated by subtracting the remaining amounts in the supernatant from the initial amounts.

To explore the adsorption mechanisms of flavin molecules on goethite surface, ATR-FTIR spectroscopy was performed using an FTIR spectrometer (Tensor II, Bruker Co., Germany) equipped with a horizontal attenuated total reflectance cell with a 45° ZnSe ATR crystal (PIKE Tech). The crystal surface was coated with a goethite film (1 mg) by drying 1 ml of 1 g L^−1^ dispersed goethite suspension at 50°C for 4 h under an N_2_-atmosphere, following a previous report (Elzinga et al., 2012). Background solution was passed through the flow cell at 2 ml min^−1^ until equilibrium, and the spectrum was used as the background spectrum. FMN or riboflavin was then injected, and the spectra were recorded in intervals of 10 min. To assist with and constrain the interpretation of flavin adsorption data, the spectra of FMN and riboflavin solution on ZnSe surface were obtained as well. All the spectra were collected using 256 scans at 4 cm^−1^ resolution.

To further assess the sequential order of IR bands in the collected IR spectra, 2D-COS analysis was performed using 2Dshige software version 1.3 (Kwansei-Gakuin University, Japan) to generate synchronous and asynchronous maps. The order of intensity change between bands υ_1_ and υ_2_ can be judged by the sign of synchronous and asynchronous correlation peak [Φ (υ_1_,υ_2_) and Ψ (υ_1_,υ_2_)], based on Noda’s rules (Noda and Ozaki, 2005). Synchronous spectrum represents the similarity of the changes in the spectral intensity observed at two wavenumbers (υ_1_ and υ_2_) along the perturbation variable *t*. When the spectral intensity changes of the two variables are (1) completely consistent, Φ reaches a positive maximum; (2) completely opposite, Φ reaches a negative maximum; and (3) irrelevant, Φ is zero. The asynchronous correlation spectrum represents the difference of the changes in the spectral intensity observed at two wavenumbers (υ_1_ and υ_2_) along the perturbation variable *t*. When Φ and Ψ are both positive or both negative, the change at υ_1_ occurs earlier than the change at υ_2_, otherwise, the change at υ_1_ occurs later.

### Confocal Laser Scanning Microscope (CLSM)

We used CLSM to analyze the bacterial attachment trend through a parallel plate flow chamber. The cover of the flow chamber was a microscope glass slide (25 mm wide, 75 mm long, and 1 mm thick) with the inner surface coated by goethite. The coating method was similar to that described above for ZnSe crystal coating. Deoxygenated HEPES buffer containing sodium formate (10 mM), goethite (~1,125 μM), and FMN or riboflavin (100 μM) was circulated by a peristaltic pump at 2 ml min^−1^ for 4 h. Then bacterial suspension (final concentration ~10^8^ cells ml^−1^) was spiked and equilibrated for 4 h. A relatively high concentration of bacterial cells was used to facilitate the observation of bacterial adhesion behavior. To further observe the effect of the interaction between FMN and goethite on bacterial adhesion, the treatments with low concentration of FMN (1 and 10 μM) were also prepared. The glass slide was removed and washed by HEPES to eliminate non-adhering bacterial cells. The attached bacterial cells were treated with LIVE/DEAD BacLight staining kit and imaged using CLSM (LSM 700, Zeiss). Twenty-four 319.53-μm^2^ images were obtained from three replicates. The density of adhering bacterial cells on goethite film was analyzed using image analysis software (Image J, NIH).

## Results

### Physicochemical Properties of *S. decolorationis* S12 and Goethite

Selected surface properties of *S. decolorationis* S12 and goethite (before and after the addition of flavins) are shown in Table 1. The presence of FMN and riboflavin did not significantly change the particle size and specific surface of goethite. When goethite reacted with FMN, the zeta potential decreased from −45.898 ± 1.327 to −56.841 ± 3.393 mV.

**Table 1:** Zeta potentials, BET surface areas, and sizes of bacteria cell and goethite before and after flavin adsorption.

Particle	Zeta potential (mV)	BET surface area (m^2^ g^−1^)	Size distribution mean (nm)
*S. decolorationis* S12	−41.791 ± 1.535	–	1,352
Goethite	−45.898 ± 1.327	81.276 ± 3.1	398 ± 30.6
Goethite + FMN	−56.841 ± 3.393	80.679 ± 1.1	362 ± 23.2
Goethite + riboflavin	−47.936 ± 3.487	80.131 ± 2.2	413 ± 29.1

*Zeta potentials were measured at 28°C and pH 7.0*.

### Comparison of the Reduction Rates of Flavin Mononucleotide and Riboflavin by *S. decolorationis* S12

**Figure 1:**
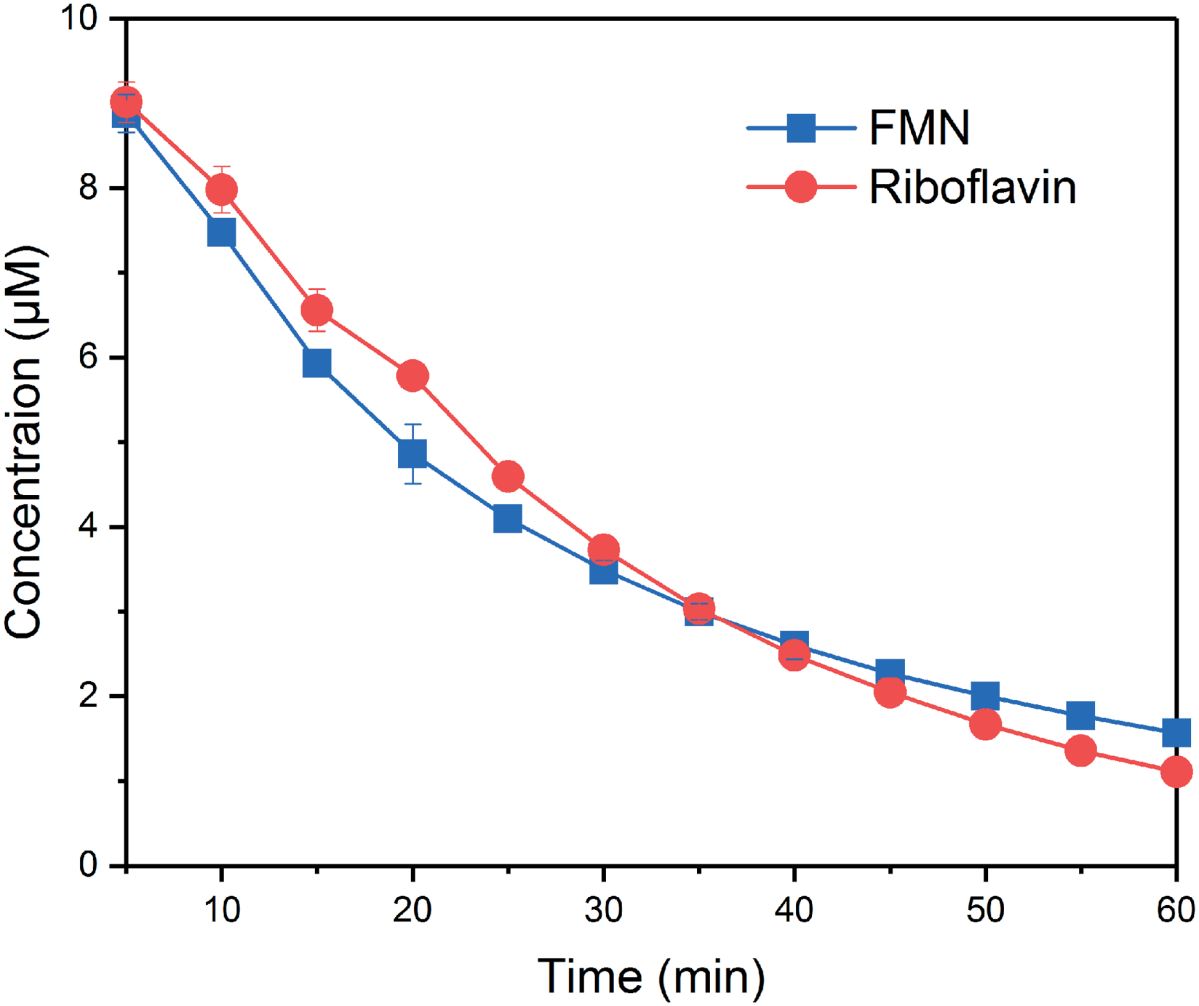
Reduction kinetics of FMN and riboflavin by *S. decolorationis* S12.

FMN and RF fluoresce when oxidized but not when reduced, which makes it possible to monitor the redox state of FMN and riboflavin. In the systems without bacteria cells, the concentration of FMN and RF did not change over time, indicating that the reduction processes were caused by bacteria cells. Figure 1 shows the time courses of FMN and riboflavin in the systems with *S. decolorationis* S12 during 60-min incubation period. FMN and riboflavin decreased quickly and remained at similar concentration level in oxidation state (1.56 ± 0.03 and 1.11 ± 0.05 μM) after incubation. In our microbial reduction experiments, only electron donor and acceptor were provided and little cell growth occurred. Under these conditions, the first-order kinetic equation is often used to describe the microbial conversion of substrates (Bosma et al., 1997; Yu et al., 2009). The kinetic constants of FMN and riboflavin reduction were 0.0374 ± 0.0011 and 0.0379 ± 0.0023 min^−1^, respectively. These results suggest that there was no significant difference in the reduction rates of FMN and RF by *S. decolorationis* S12 (*p* > 0.05).

### Comparison of the Reduction Rates of Goethite by FMNH_2_ and RFH_2_

**Figure 2:**
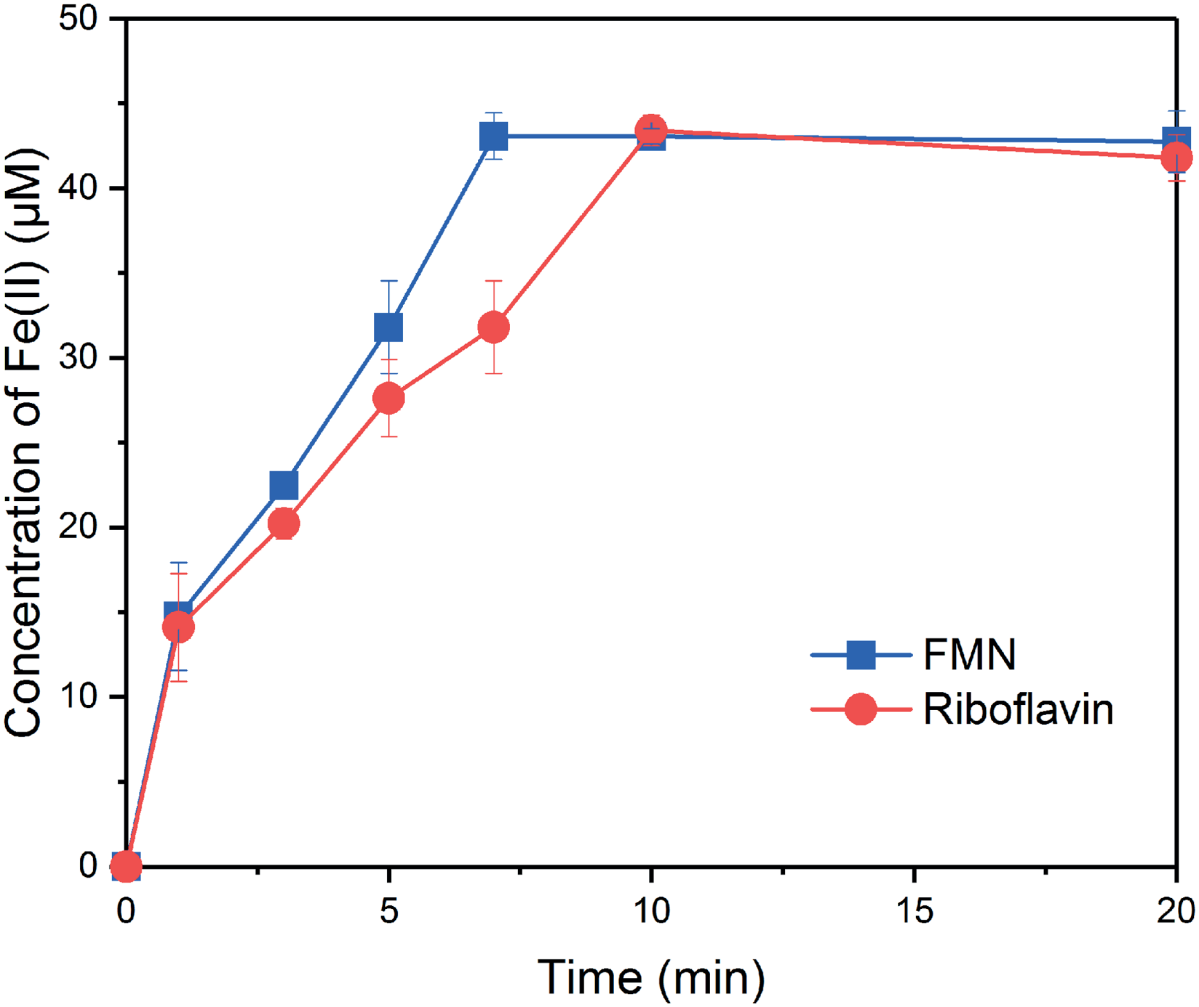
Reduction kinetics of goethite by FMNH_2_ and RFH_2_.

In order to evaluate the ability of FMNH_2_ and RFH_2_ (reduced forms of FMN and riboflavin) to reduce goethite, the reductive kinetics of goethite by FMNH_2_ and RFH_2_ were investigated. The results showed that both FMNH_2_ and RFH_2_ could reduce goethite and the reactions were completed in ~7 min (Figure 2). At the end of reduction experiment, approximately 42 μM Fe(II) was produced. The difference from the expected 44 μM might result from the adsorption of Fe(II) onto goethite (Urrutia et al., 1999). The first-order reduction rate constants for FMNH_2_ and RFH_2_ were 0.29 ± 0.02 and 0.22 ± 0.04 min^−1^, indicating that FMNH_2_ reduced goethite faster than RFH_2_.

### Reduction of Goethite by *S. decolorationis* S12 in the Presence of Different Concentrations of Flavin Mononucleotide and Riboflavin

**Figure 3:**
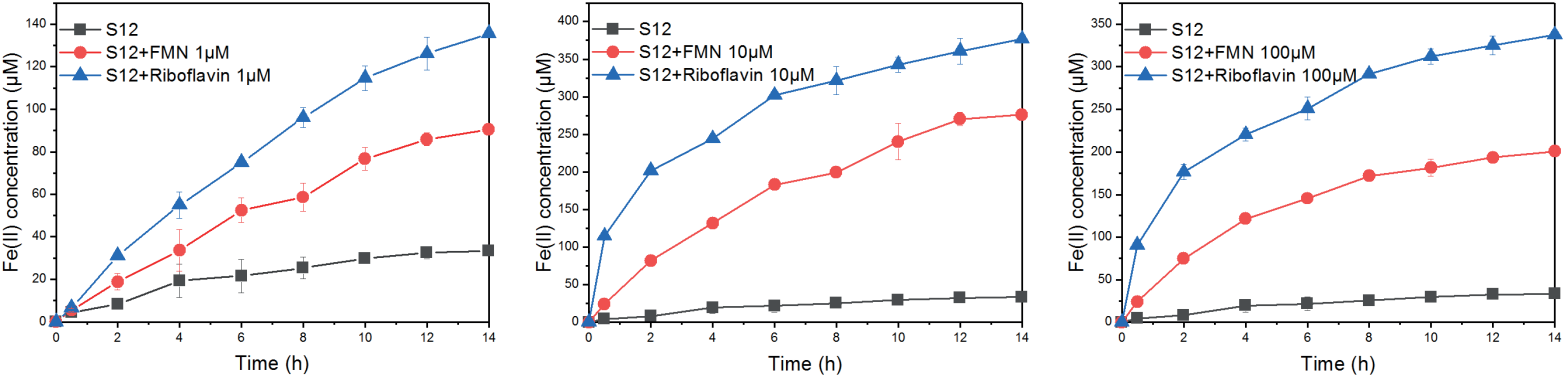
Reduction kinetics of goethite by *S. decolorationis* S12 at different concentrations of FMN and riboflavin.

The bioreduction of goethite in the presence of different concentrations of FMN and riboflavin is shown in Figure 3. For flavin-free treatment, only a small amount of ferrous iron (33.32 ± 0.79 μM) was observed at 14 h, indicating that the reduction rate of goethite by *S. decolorationis* S12 was slow. Compared with flavin-free treatment, the first-order reduction rate constants in the presence of 1, 10, and 100 μM of FMN increased by 2.07-, 8.83-, and 5.27-fold, respectively. The counterparts for riboflavin treatment were 3.65-, 9.55-, and 8.62-fold. These results suggest that FMN and riboflavin enhanced the bioreduction of goethite by *S. decolorationis* S12, and that riboflavin outperformed FMN for the concentration range of 1–100 μM.

### The Behavior of Flavin Mononucleotide and Riboflavin on the Surface of Goethite

**Figure 4:**
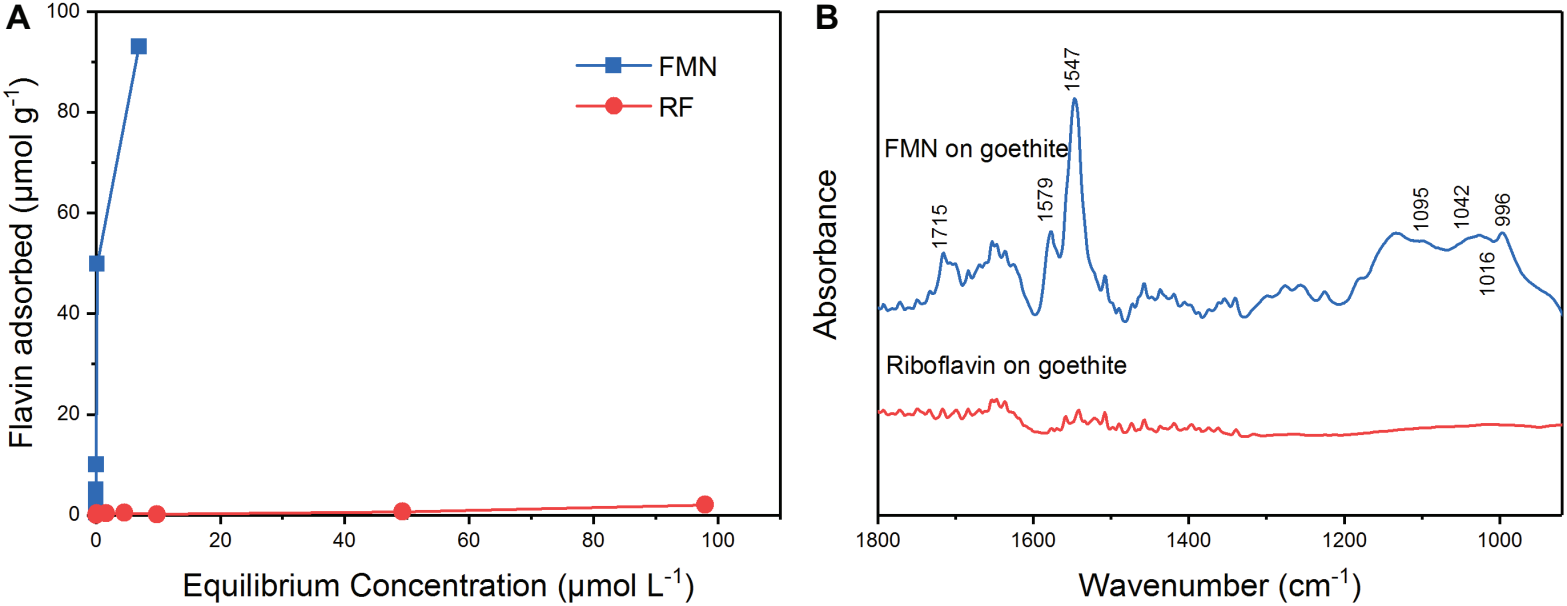
Sorption of FMN and riboflavin on goethite **(A)**; ATR-FTIR spectra of FMN and riboflavin on goethite **(B)**.

The results of the isothermal adsorption experiment are shown in Figure 4. The amount of adsorption of FMN on goethite increased with the equilibrium concentration. When the initial concentration of FMN ranged from 0.5 to 50 μM, the equilibrium concentration was close to 0 μM (Figure 4A). The adsorption data conformed to the Langmuir model and the maximum adsorption capacity of FMN on goethite was 95.88 ± 3.09 μmol g^−1^. For riboflavin, the adsorption quantity did not change significantly as the equilibrium concentration increased, indicating that little adsorption occurred. The results showed that goethite surface adsorbed FMN in large amounts, but the amount of adsorbed riboflavin was very limited.

In order to identify the molecular mechanism of FMN binding to goethite surface, *in situ* ATR-FTIR spectroscopy was used to explore FMN interfacial behavior on the goethite surface. As shown in Figure 4, two regions were observed on the spectrum of FMN on goethite: one was the isoalloxazine ring region (1,700–1,500 cm^−1^), and the other was phosphate group region (1200–900 cm^−1^). Consistent with the previous studies, the bands at 1715 cm^−1^ could be assigned to C(4)═O stretching vibration, and 1,579 and 1,547 cm^−1^ originate mainly from C(4a)═N(5) and C(10a)═N(1) stretching vibrations of the isoalloxazine ring (Bowman and Spiro, 1981; Iwata et al., 2006; Spexard et al., 2011). In the region of phosphate groups, several vibrations were resolved, including 1,095 cm^−1^ [υ_s_ (P═O)], 1,042 cm^−1^ and 1,016 cm^−1^ (υ P─OFe), and 996 cm^−1^ [υ_as_ P─(OFe)_2_] (Tejedortejedor and Anderson, 1990; Parikh and Chorover, 2006; Wei et al., 2016). This result indicated that the P-moiety in FMN molecular formed inner-sphere complex with the surface of goethite, which was similar to the previous reports regarding P─Fe (hydr)oxide interaction (Tejedortejedor and Anderson, 1990; Elzinga and Kretzschmar, 2013). Compared with the FMN spectrum, there was no significant adsorption band in the range of 1800–900 cm^−1^ in the spectrum of riboflavin on goethite.

**Figure 5:**
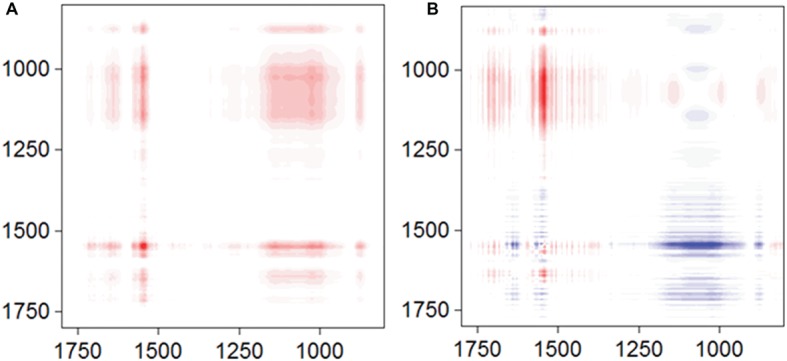
Synchronous **(A)** and asynchronous **(B)** contour maps obtained from the time-dependent ATR-FTIR spectra of FMN sorbed on goethite for 4 h equilibration.

To obtain more information about the binding process, 2D-COS analysis was conducted using a set of time-dependent FTIR spectra, and the synchronous and asynchronous maps are shown in Figure 5. In the synchronous map, all autopeaks were positive, implying that the observed IR peaks change simultaneously during the adsorption process. In the asynchronous map, the main cross peaks at the isoalloxazine ring/phosphate group region (1,715, 1,579, and 1547/996 cm^−1^) were positive. According to Noda’s rule, the sequence of the spectral change at 996 cm^−1^ was later than the other cross peaks, indicating that the inner-sphere complex formed after the isoalloxazine ring contacted the surface of goethite.

### The Effect of Flavin on the Behavior of *S. decolorationis* S12 on Goethite Surface

**Figure 6:**
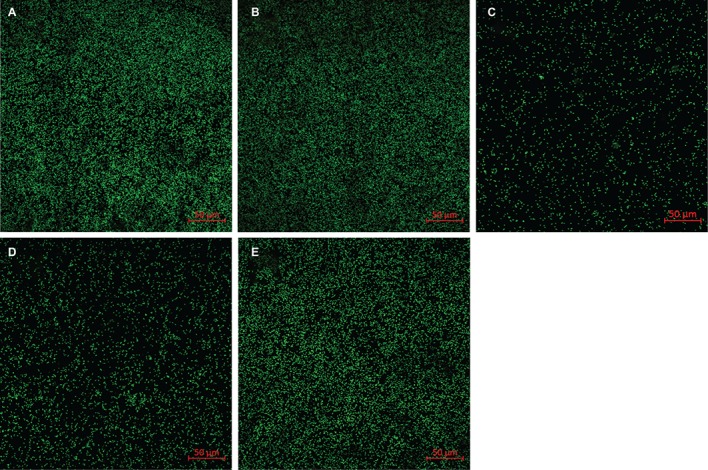
Representative CLSM images of *S. decolorationis* S12 on goethite **(A)**, goethite + riboflavin (100 μM) **(B)**, goethite + FMN (100 μM) **(C)**, goethite + FMN (10 μM) **(D)** and goethite + FMN (1 μM) **(E)** after 4 h. The area of each image is 319.53 μm^2^. The surface cell density was 0.089 ± 0.003, 0.088 ± 0.004, 0.020 ± 0.006, 0.029 ± 0.006, and 0.076 ± 0.008 cells μm^−2^, respectively.

To explore whether the adsorption of flavin on the surface of goethite affects the interaction of bacterial cells with goethite, we observed bacterial behavior on goethite surface using CLSM (Figure 6). The surface cell density for flavin-free, FMN (100 μM), and riboflavin (100 μM) treatments was 0.089 ± 0.003, 0.020 ± 0.006, and 0.088 ± 0.004 cells μm^−2^, respectively. The number of bacterial cells attached on the surface of goethite after 100 μM FMN treatment was significantly reduced, whereas riboflavin did not affect bacterial adhesion. Moreover, the number of attached bacteria in the presence of 1 and 10 μM FMN was also lower than that of the control and riboflavin treatment (Figure 6).

## Discussion

FMN and riboflavin are structurally similar flavins, except for the presence of a phosphate group on the FMN molecule. In our study, the enhancement of goethite bioreduction by FMN was weaker than that in the case of riboflavin, indicating that the phosphate group in FMN molecule hindered flavin-regulated extracellular electron transfer from bacterial cells to goethite surface. However, there was no difference in the bacterial reduction rate of FMN and riboflavin, suggesting that the phosphate group does not affect electron transfer from bacterial cells to the isoalloxazine ring. In addition, FMNH_2_ reduced goethite quicker than RFH_2_, consistent with the results of a previous report (Shi et al., 2013). The data of adsorption experiment, ATR-FTIR, and 2D-COS analysis showed that the isoalloxazine ring with C(4)═O, C(4a)═N(5), and C(10a)═N(1) groups contacts goethite surface before forming phosphate inner-sphere complex (Figures 4B, 5). Therefore, the incoming or outgoing electrons of N(1) and N(5) in the isoalloxazine ring of FMN may not be hindered by the phosphate group.

The bacterial cells are the source of electrons, and approximately 90% of the reductive activity of FMN and riboflavin occurs at *Shewanella* outer membrane using MtrC and OmcA (Coursolle et al., 2010). Therefore, goethite closer the bacterial outer membrane would be more susceptible to accept electrons from FMNH_2_. Furthermore, MtrC and OmcA can specifically associate with FMN and riboflavin (Okamoto et al., 2013). The interaction between flavin and outer-membrane hemeprotein complex results in a faster reaction rate (by 10^3^–10^5^ folds) than that of free flavin (Okamoto et al., 2013). Previous research has shown that flavin-dependent extracellular electron transfer is mediated mainly by outer-membrane hemeproteins-bound flavins (Xu et al., 2016). Therefore, goethite in contact with the outer membrane would be reduced more quickly.

CLSM analyses showed that FMN significantly reduced the number of bacterial cells attached to the goethite surface compared with riboflavin (*p* < 0.05) (Figure 6). The adsorption experiment and ATR-FTIR analysis indicated FMN molecules adsorb to goethite surface by forming the inner-sphere bond between the phosphate group and goethite surface. Previous research found that the interaction of phosphate with FeOOH particle surfaces results in a 75% reduction in the adhesion of *E. coli* on the mineral surface (Appenzeller et al., 2002). In addition, surface-bound FMN increased the negative charge on goethite (Table 1), which would increase the electrostatic repulsion between goethite and the negatively charged *S. decolorationis* S12 cells. Therefore, the interaction between FMN and goethite may prevent bacterial cells from approaching goethite, which leads to an increase in the distance between bacterial outer membrane and goethite surface. The rate of FMN-mediated electron transfer from bacterial outer membrane to goethite may be lower than that mediated by RF.

**Figure 7:**
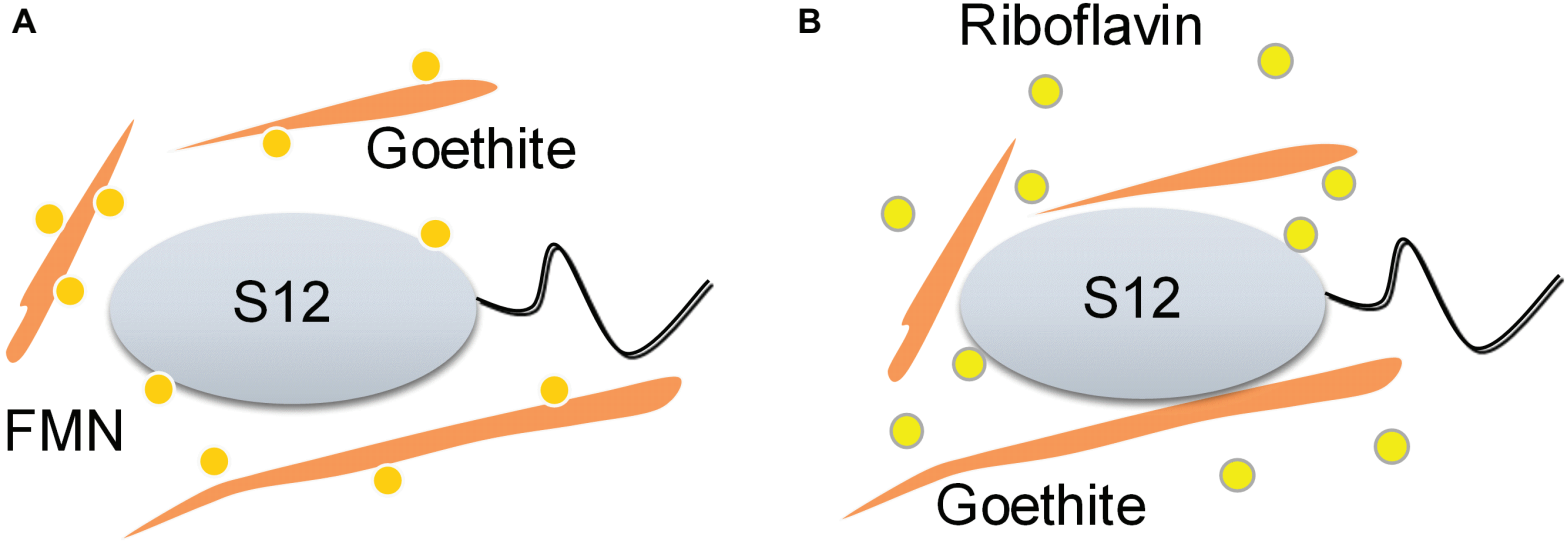
Schematic of the proposed effect mechanisms of flavin/goethite interaction on the reduction of goethite by *S. decolorationis* S12. FMN exhibits strong adsorption on goethite surface by forming phosphate inner-sphere complex, which prevents bacterial cells from approaching goethite and may increase the distance of electron transfer from bacterial cells to goethite **(A)**. As for riboflavin, the interaction between riboflavin and goethite was weak and did not affect the bacterial behaviors on the surface of goethite **(B)**.

Our study provides the first insights into the role of flavin/Fe (hydr)oxide interaction in flavin-mediated microbial Fe reduction. Previous research on flavin recognized that FMN and riboflavin could be associated with outer-membrane hemeproteins, but overlooked the effect of flavin/iron (hydr)oxide interactions. Here, we found that the interaction of FMN and goethite can affect bacterial behavior on the surface of goethite, thereby, interfering with the process of extracellular electron transfer to the mineral surface (Figure 7).

## Data Availability

All datasets generated for this study are included in the manuscript and/or the supplementary files.

## Author Contributions

GZ and MX designed the study. GZ and EL operated the experiments. JL, FL, and XY discussed the results. GZ and MX wrote the paper. All authors agree to be accountable for the content of the work.

### Conflict of Interest Statement

The authors declare that the research was conducted in the absence of any commercial or financial relationships that could be construed as a potential conflict of interest.
